# Comparison of the clinical and microbiological characteristics of *Campylobacter* and *Helicobacter* bacteremia: the importance of time to blood culture positivity using the BACTEC blood culture systems

**DOI:** 10.1186/s13104-017-2981-2

**Published:** 2017-11-28

**Authors:** Kei Yamamoto, Kayoko Hayakawa, Maki Nagashima, Kayo Shimada, Satoshi Kutsuna, Nozomi Takeshita, Yasuyuki Kato, Shuzo Kanagawa, Koji Yamada, Kazuhisa Mezaki, Teruo Kirikae, Norio Ohmagari

**Affiliations:** 1 Disease Control and Prevention Center, National Center for Global Health and Medicine, 1-21-1 Toyama, Shinjuku-ku, Tokyo, 162-8655 Japan; 2 Department of Infectious Diseases, Research Institute, National Center for Global Health and Medicine, 1-21-1 Toyama, Shinjuku-ku, Tokyo, 162-8655 Japan; 3 Clinical Laboratory, National Center for Global Health and Medicine, 1-21-1 Toyama, Shinjuku-ku, Tokyo, 162-8655 Japan

**Keywords:** *Helicobacter*, *Campylobacter*, Spiral-shaped bacilli, Bacteremia, BACTEC

## Abstract

**Objective:**

*Campylobacter* spp. and *Helicobacter* spp. are rare but important causes of bacteremia in humans. Distinguishing these bacteria is complicated because of their similar phenotypic profiles. We conducted clinical and microbiological investigations of *Campylobacter* spp. or *Helicobacter* spp. bacteremia. Patients diagnosed with bacteremia from 2008 to 2014 were included. The clinical and microbiological characteristics of *Campylobacter* spp. and *Helicobacter* spp. bacteremia were compared. The BACTEC system was used in blood cultures. A receiver operating characteristic curve was plotted based on the time to blood culture positivity.

**Results:**

Sixteen cases of *Helicobacter* spp. bacteremia (patient age: 61 ± 18 years) and 14 cases of *Campylobacter* spp. bacteremia (patient age: 49 ± 21 years) were identified. Median time to blood culture positivity was longer for the *Helicobacter* spp. cases than the *Campylobacter* spp. cases (91.4 h vs 55.3 h, *p* < 0.01). A time to blood culture positivity > 75 h predicted *Helicobacter* spp. bacteremia with a sensitivity of 0.88 and a specificity of 0.93 (area under the receiver operating characteristic curve of 0.90). In conclusion, a time to blood culture positivity was useful in distinguishing *Helicobacter* spp. bacteremia from *Campylobacter* spp. bacteremia.

## Introduction

Bacteremia due to enterohepatic *Helicobacter* species (HS) has frequently been reported [1–6]. Because HS resembles *Campylobacter* species (CS) on Gram staining, it is difficult to distinguish from CS based on positive blood cultures. Accordingly, HS have often been misidentified as CS [6]. Previous reports have noted substantial differences between HS and CS in terms of antibiotic susceptibility and clinical courses [2, 7, 8]; thus, microbiological identification is crucial to improve patient care. Although the use of matrix-assisted laser desorption/ionization time-of-flight mass-spectrometry (MALDI-TOF) can help distinguish HS from CS by direct analysis of individual cultured colonies [9], HS are usually difficult to culture, and these species take about 2–3 days to grow. MALDI-TOF was used in directly identifying blood culture isolates from positive blood cultures [10]. However, no study on the identification of HS and CS on positive blood culture broths using MALDI-TOF is available. A simple predictor is required in distinguishing HS from CS when blood cultures are positive of spiral-shaped bacteria. We conducted clinical and microbiological investigations of these two bacteremia, and compared HS with CS in terms of time to blood culture positivity (TTBP) to obtain a TTBP cut-off value that could be used to predict HS bacteremia.

## Main text

### Materials and methods

#### Patients and definitions

The study used a retrospective, single-center, and case control design. The medical records of all patients at the National Center for Global Health and Medicine (779 beds, Tokyo, Japan) who had bacteremia due to HS or CS were reviewed between January 2008 and April 2014. We collected the following clinical information: age at diagnosis, sex, history of animal contact, hospital-acquired infections, underlying diseases, side effects of immunosuppressants, clinical manifestations, antibiotic treatments, and outcomes. We defined “persistent bacteremia” as constant positive blood cultures 48 h after the start of antibiotics. “Recurrence” was defined as the recurrence of bacteremia caused by the same species after the completion of an initial antibiotic course. Furthermore, cases in which the patient had stayed at the hospital for more than 2 days prior to bacteremia were classified as “hospital-acquired infections.”

#### Bacterial isolates

All blood culture samples were collected into standard aerobic and anaerobic culture bottles (92F, 93F, Becton–Dickinson Microbiology Systems, Sparks, MD, USA) and processed using the BACTEC 9240 or 9120 systems (Becton–Dickinson Microbiology Systems, Sparks, MD, USA). These samples were routinely monitored for at least 120 h (from January 2008 to March 2009) or 144 h (from April 2009 to April 2014). If a physician required a prolonged incubation of the blood culture, the duration was extended to 21 days (within 504–528 h). Because blood culture samples with extended incubation time were manually monitored manually and routinely checked on the 14th and 21st days, the exact TTBP in these cases cannot be determined. Instead, the TTBP was set to 144 h if the samples were positive during the extended incubation time.

A microaerobic culture was performed using modified Skirrow agar (Nissui Pharmaceutical Co., Ltd., Tokyo, Japan) and Chocolate agar (Kyokuto Pharmaceutical Industrial Co., Ltd., Tokyo, Japan) at 35 °C, using AnaeroPack for a microaerophilic environment (Mitsubishi Gas Chemical Company, Tokyo, Japan). When the bacteria could not grow on modified Skirrow agar, we used tryptic soy agar/broth with 5% sheep blood (own mixture) at 35 °C for 7 days. The grown colonies were identified by using API Campy systems (bioMérieux, Marcy l’Etoile, France), which can distinguish HS from CS through biochemical characterization.

#### DNA preparation and strain identification through 16S rRNA sequencing

The subspecies were further identified through molecular analysis under these two conditions: if no colony grew on the agar or if sheet-shaped colonies grew, which were suspected to be HS. DNA was extracted from fresh colonies grown on modified Skirrow agar using the hot extraction method. Bacterial 16S rRNA gene sequencing was performed as previously described [11], using the following primers: 5F (5′-TTG GAG AGT TTG ATC CTG GCT C-3′) and 1194R (5′-ACG TCA TCC CCA CCT TCC TC-3′) between January 2008 and March 2013 or 5F and 1485R (5′-TAC GGT TAC CTT GTT ACG AC-3′) between April 2013 and April 2014. Amplicon sequencing was performed using the following primers: 5F and 810R (5′-GGC GTG GAC TTC CAG GGT ATC T-3′) between January 2008 and March 2013 or 341A (5′-CTA CGG GAG GCA GCA GTG GG-3′), 519B (5′-ATT ACC GCG GCK GCT G-3′), 907A (5′-AAA CTY AAA KGA ATT GAC GG-3′), and 1194R between April 2013 and April 2014. Electrophoresis was performed using an ABI 3130 system (Life Technologies, Carlsbad, CA, USA). The sequence obtained was compared with all known sequences in GenBank by using the online database’s Basic Local Alignment Search Tool (BLAST, National Center for Biotechnology Information [http://blast.ncbi.nlm.nih.gov]).

#### Statistical analysis

Fisher’s exact test or the Mann–Whitney U test was carried out to compare the characteristics of HS and CS. The ROC curve was plotted, and the area under the ROC curve (AUC) was calculated using SPSS (version 24; IBM, New York, USA). The cut-off value was calculated by maximizing Youden’s index (sensitivity + specificity − 1) [12].

### Results

#### Clinical characteristics

During this study period, 62,073 blood culture sets, of which 3028 sets were collected from children, were tested in our hospital. Fifty-seven (0.09%) blood cultures placed inside the bottles with aerobic organisms were positive of spiral-shaped bacilli. Thirty patients were diagnosed with bacteremia based on the presence of spiral-shaped bacilli. All patients’ characteristics and outcomes are shown in Table 1. The most common underlying disease was solid organ cancer, particularly lung cancer. Skin lesions were common in HS bacteremia, whereas diarrhea was a common symptom of CS bacteremia. Other rare infectious clinical syndromes included graft vessel infection due to *H. cinaedi/H. bilis*, cellulitis due to *C. jejuni*, and acute obstructive cholangitis due to *C. jejuni*.

**Table 1:** Patient characteristics and outcomes of *Campylobacter and Helicobacter* bacteremia

	*Helicobacter* spp.	*Campylobacter* spp.
N	16	14
Age (years), mean ± SD	61 ± 18	49 ± 21
Sex (male)	10	8
Hospital-acquired infection*	7 (43.8%)	1 (7.1%)
Animal contact	3 (18.8%)	1 (7.1%)
Underlying diseases	16 (100%)	10 (71.4%)
Solid organ cancer	8	4
Hematological malignancy	4	1
Liver cirrhosis	4	2
Collagen vascular disease	2	1
Others	2	5
Side effects of immunosuppressants (including chemotherapy for cancer)*	8 (50%)	1 (7.1%)
Clinical manifestations		
Fever (> 37.5 °C)	11	13
Diarrhea^†^	0	7
Skin lesion*	8	1
Arthralgia	0	3
Headache	2	3
Others	2	5
No apparent symptom	5	1
*Body temperature (°C), mean ± SD	38.1 ± 1.0	38.8 ± 0.9
Treatment		
No antibiotics	1	1
Penicillin	11	2
Cephalosporin	9	7
β-lactamase/β-lactam	4	4
Carbapenem	5	3
Macrolide	2	2
Fluoroquinolone	2	3
Doxycycline/minocycline	12	2
Others	0	1
Total duration of antibiotic treatment (days),mean ± SD^†^	36 ± 26	10 ± 8
Persistent bacteremia (> 48 h)	2	0
Recurrent case	3	0

* *p* value < 0.05, ^†^ *p* value < 0.01

Twenty-eight (93%) patients were treated with antibiotic, of which 25 patients (89%) received antibiotics before identification of the bacteria. During the initial therapy, 93% of the patients were treated using monotherapy, particularly with beta-lactam agents or carbapenem. The combination therapy of amoxicillin and doxycycline was used in 10 patients with HS bacteremia (62.5%). The duration of antibiotic use was significantly longer for patients with HS bacteremia those with CS bacteremia (*p* < 0.001). Two patients had persistent HS bacteremia despite antibiotic treatment, and 3 patients experienced recurrence of HS bacteremia (Table 2). These 3 patients did not experience the third recurrence. Five patients with HS and 3 patients with CS died during the study period. However, none of these patients died because of infection-related causes.

**Table 2:** Recurrent and persistent cases of bacteremia due to *Helicobacter* spp.

No.	Age sex	Underlying diseases	Clinical manifestations	Immunosuppressants	Initial ABXs (days)	Duration from discontinuation of ABXs to recurrence (days)	ABX regimens after re-starting or changing the ABXs	Total duration of ABXs (days)
Recurrence								
1	40s Male	Small bowel obstruction Chronic diarrhea	Skin lesion	None	SAM (13) >AMX (14)	8	CRO AMX + DOX	115
2	70s M	Lung adenocarcinoma Metastatic lesion	FN	Gefitinib Carboplatin Pemetrexed	SAM (4) >FEP (16)	11	FEP AMX + DOX	41
3	50s Male	Lung ewing sarcoma	FN Skin lesion	Vincristine Doxorubicin Cyclophosphamide Ifosfamide Etoposide	FEP (10)	13	FEP AMX + DOX	51
Persistent bacteremia								
1	60s Male	Tibial plateau fracture (after operation)	Skin lesion Joint infection	None	CFZ (11)	–	MEM DOX	85
2	80s Female	Osteoarthritis (after TKA) Hodgkin disease (complete remission)	Skin lesion Joint infection	None	CLR (19)	–	MEM AMX + DOX	89

*FN* febrile neutropenia, *ABXs* antibiotics, *TKA* total knee arthroplasty, *SAM* ampicillin sulbactam, *AMX* amoxicillin, *CRO*, ceftriaxone, *DOX* doxycycline, *FEP* cefepime, *CFZ* cefazolin, *MEM* meropenem, *CLR* clarithromycin

#### Microbiological characteristics

**Fig. 1:**
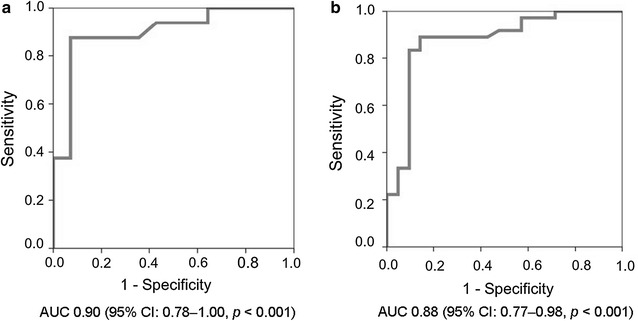
ROC curve demonstrating the TTBP used to diagnose bacteremia caused by spiral-shaped bacteria. ROC obtained from the results that were positive for spiral-shaped bacteria, of the first isolation from each patient (**a**) and those of all isolations (**b**) for TTBP to diagnose bacteremia due to *Helicobacter* spp. AUC, area under the receiver operating characteristic curve

Of 57 blood cultures, 36 were positive for HS and 21 were positive for CS. The HS were *H. cinaedi* (n = 30), *H. fenneliae* (n = 4), or undetermined (n = 2). The CS were *C. jejuni* (n = 17), *C. coli* (n = 2), or undetermined (n = 2). Of the four undetermined species, 1 HS could not be determined as *H. bilis* or *H. cinaedi* through 16 s rRNA sequencing. Only 1 HS and 2 CS were examined using the API Campy systems, and their species level was not identified. We analyzed the results of the first isolation from each patient. For 4 patients (3 infected with HS and 1 with CS), the delay between the collection of blood samples and the start of the incubation exceeded 24 h. The median TTBP was longer in HS cases (91.4 h; inter-quartile range [IQR]: 80.4–122.1) than CS cases (55.3 h; IQR: 50.3–67.6) (*p* < 0.01). For 1 patient with HS bacteremia, the blood culture was prolonged beyond 144 h. As a means of predicting HS bacteremia, TTBP had an AUC of 0.90 (95% confidence interval [CI] 0.78–1.00, *p* < 0.001) (Fig. 1a). Because Youden’s index used the maximum of 74.1 h, we established the cut-off value as 75 h. A TTBP value > 75 h predicted HS bacteremia with a sensitivity of 0.88 and a specificity of 0.93. In a subsequent analysis of the results of all isolations, we found that TTBP had an AUC of 0.88 (*p* < 0.001) for the prediction of HS bacteremia (Fig. 1b). Additionally, TTBP > 75 h predicted HS bacteremia with a sensitivity of 0.83 and a specificity of 0.91.

### Discussion

The TTBPs of HS and CS significantly varied in our analyses of both the initial and all isolations included in our study. TTBP was a good parameter for distinguishing these 2 types of spiral-shaped bacteria. A 5-day median TTBP for *H. cinaedi* was reported in a recent study that used a BACTEC system for blood cultures [1]. Additionally, the median TTBP for *C. jejuni* was previously reported to be 5 days [13], which is longer than the TTBP in our study (median 2.3 days). Differences in the results of the present study may be due to the blood culture of *C. jejuni* that was implemented using an older BACTEC system as previously reported. Although TTBP was a good predictor for HS or CS bacteremia when blood culture becomes positive, MALDI-TOF is still among the most rapid and accurate identification methods. Grown bacterial colonies are used in identifying the type of bacteria using MALDI-TOF, and Winkler et al. reported that this method was useful for HS and CS [9]. However, bacterial colonies of both HS and CA take about 2–3 days to grow. HS was more difficult to grow on medium than CS [7]. Similarly, more than half of the HS were not grown in our study (data not shown). Several studies about the identification of microorganisms using MALDI-TOF from direct positive blood culture broths [10] or direct positive blood culture subsequent to short incubation on solid medium are available [14]. Although HS may be distinguished from CS when blood culture becomes positive, the efficacy of these methods used in identifying HS and CS have not been verified. Moreover, microbiological laboratories in several hospitals do not use MALDI-TOF because of its cost. In conclusion, a TTBP cut-off of > 75 h was useful in distinguishing bacteremia due to HS from bacteremia due to CS when using the BACTEC systems.

Although there are no guidelines for the treatment for HS bacteremia, many antibiotic agents have been used successfully, both alone and in combination [7]. Although the breakpoint of *H. cinaedi* has not been established, it has been reported that the MICs of tetracyclines, carbapenems, and aminoglycosides are relatively low, and that those of ampicillin and cephalosporins are moderate. In contrast, MICs of macrolides and fluoroquinolones are relatively high for *H. cinaedi* [3, 4, 7]. Unfortunately, drug susceptibility testing for *Helicobacter* spp. could be performed for only one strain that was isolated from the patient without recurrent or persistent bacteremia. It was only possible to test a single strain because it was difficult to culture *Helicobacter* spp. on the plate and to interpret the susceptibility test result. In all cases of recurrent or persistent HS bacteremia, patients had only been treated with beta-lactam agents prior to recurrence or persistent bacteremia. Although we suspect that the MICs of the antibiotics were related to the prognosis of HS, further studies are needed in order to clarify this relation.

## Limitations

This study had two major limitations. First, our assessment of TTBP as a means of distinguishing HS from CS is only applicable to the BACTEC systems. Other automated blood culture systems, such as the BacT/Alert system, have early HS TTBPs and lower detection rates than the BACTEC systems [7]. Consequently, the TTBPs of HS and CS may differ from the results of our study when other automated blood culture systems are used. Second, *C. fetus* was not detected in our institute. The clinical manifestations of bacteremia due to *C. fetus* differ from those of bacteremia due to other CS [8]. Therefore, the clinical manifestations that were reported in the present study may differ from those of other institutes where *C. fetus* is frequently detected. However, because *C. fetus* do not have a longer TTBP than other CS, the usefulness of TTBP in distinguishing spiral-shaped bacteria may not be affected.

## Abbreviations

HS: *Helicobacter* species
CS: *Campylobacter* species
MALDI-TOF: matrix-assisted laser desorption/ionization time-of-flight mass-spectrometry
TTBP: time to blood culture positivity
AUC: area under the ROC curve
